# Looking Ahead

**Published:** 1889-05

**Authors:** 


### LOOKING AHEAD.

Now that the regular college year has ended in most of our institutions, and the less arduous duties of the spring course are on hand, we desire to call the attention of the several dental faculties which send representatives to the National Association of Dental Faculties, to two resolutions that were passed at its last session regarding the proposed lengthening of the college term and the requirements for a seat and voice in the coming session of the association to be held at Saratoga, in August next. At the last meeting a motion was offered by Dr. Brophy and carried by the association, to the effect that "hereafter a delegate representing a college of the association shall be a member of the teaching faculty of such college, and shall present properly executed credentials specifying his authority to represent his college, before he shall be allowed to vote on questions before the association."

In order that the delegates should receive some definite form of instruction, Dr. Eames offered the following resolution, which was adopted: "Resolved, that it is the sense of this meeting that the course of instruction in all colleges of this association should be three years of not less than five months each; and that delegates shall submit the proposition to their respective faculties, and report their action to this association at its next annual meeting, in order that a decision on the question may be had." There was an evident feeling at the last meeting of the need for an extension of the college term, and an expressed desire upon the part of the members of the association present to take some decided step in the near future. In order that whatever action is brought forward at the session this fall may be binding upon the several institutions that are members of the association, and in order that each college shall have representation in the meeting and a voice in its proceedings, it is absolutely necessary that certified authority be given the member of the faculty who is sent to represent the college in the association. A full and interesting meeting is expected, and it is hoped that some marked step in the direction of higher education will be taken; therefore, do not fail to come prepared legally to represent your institution.

